# Investigation on care knowledge–attitude–practice of the main caregivers of patients with dysphagia following stroke

**DOI:** 10.3389/fneur.2025.1667734

**Published:** 2026-01-12

**Authors:** Mei Yu, Xiaolei Chen, Xiaodong Cao

**Affiliations:** Nursing Department, The Affiliated Wuxi People's Hospital of Nanjing Medical University, Wuxi Medical Center, Wuxi People's Hospital, Wuxi, Jiangsu, China

**Keywords:** stroke, dysphagia, the main caregiver, knowledge–attitude–practice, rehabilitation

## Abstract

**Objective:**

To investigate the status of care knowledge–attitude–practice (KAP) of the main caregivers of patients with dysphagia following stroke, analyse the influencing factors, and provide a basis for health education for clinical medical staff.

**Method:**

A total of 268 primary caregivers of stroke dysphagia who met the inclusion criteria between May 2023 and December 2024 in the departments of neurology and rehabilitation of a tertiary hospital in Jiangsu Province were recruited as the survey participants via convenience sampling. The general information of the main caregivers and their nursing knowledge, attitude, and practice were then collected for analysis.

**Results:**

Cronbach's alpha coefficient of the self-made questionnaire was 0.906, the split-half reliability was 0.939, and the scale-level content validity index was 0.95, indicating good reliability and validity. The average score of the knowledge dimension was 7.30 ± 4.07 points; the average score of the attitude dimension was 19.70 ± 3.49 points; the average score in the practice dimension was 19.56 ± 5.22 points. Multiple linear regression analysis showed that the age of the caregiver (β = −0.136, *P* = 0.014), cultural level (β = 0.485, *P* < 0.001), the treatment status of dysphagia (β = −0.108, *P* = 0.008), care time (β = 0.277, *P* < 0.001), and monthly income (β = 0.178, *P* < 0.001) were the influencing factors of knowledge–attitude–practice.

**Conclusion:**

The primary caregivers' care knowledge and behavior need to be improved, although the care attitude is more positive. Education level, care time, age of caregivers, monthly income, and treatment of swallowing disorder were the influencing factors.

## 1 Introduction

Post-stroke dysphagia refers to a clinical condition in which impairment of the central nervous system responsible for swallowing disrupts the safe transfer of food from the oral cavity to the gastrointestinal tract. It is one of the most common sequelae following stroke (1). The global incidence of post-stroke dysphagia ranges from 59 to 76% (2), with 3%−50% of patients experiencing persistent symptoms beyond 6 months (3). Currently, there are no targeted pharmacological treatments for post-stroke dysphagia, and the recovery of swallowing function primarily relies on rehabilitation therapy (4, 5). Considering the prolonged duration of dysphagia rehabilitation, high hospital bed turnover, and financial constraints, most patients continue rehabilitation at home once their condition stabilizes (6). Studies have confirmed that effective family caregiving is crucial for promoting both functional recovery and overall physical and psychological wellbeing in survivors of stroke (7). Expert consensus recommends that the rehabilitation of post-stroke dysphagia should involve a multidisciplinary team, including family caregivers (8). Given that specialized feeding techniques and dietary selection are necessary for patients with dysphagia, a lack of safe and effective caregiving knowledge and practices may expose patients to complications such as aspiration (9). International studies have shown that family caregivers of survivors of stroke often express fear of choking during meals, lack experience in managing emergencies such as airway obstruction, feel frustrated by the patient's inadequate nutritional intake, and experience anxiety due to prolonged mealtimes (10, 11). However, similar research remains limited in China. Therefore, it is essential to assess the current status of caregiving knowledge and practices among primary caregivers of patients with post-stroke dysphagia and to implement targeted health education to help reduce caregiver burden and improve patients' quality of life.

The knowledge–attitude–practice (KAP) survey model is one of the well-established frameworks for promoting healthy behavioral change. It delineates a three-stage process: acquiring knowledge, forming beliefs, and modifying behavior (12). According to this theory, the acquisition of adequate health-related knowledge facilitates the development of positive attitudes and beliefs, ultimately leading to the adoption of health-promoting behaviors. The KAP survey model has become a significant theoretical foundation in behavioral medicine and is extensively applied in clinical nursing and nursing education. Researchers can design a KAP survey tailored to specific study objectives to assess individuals' knowledge, attitudes, and practices regarding particular diseases or health issues, as well as to analyse influencing factors. This approach supports the implementation of targeted health education interventions aimed at improving knowledge, attitudes, and practices among specific populations. In recent years, the KAP framework has also been increasingly applied to evaluate the caregiving knowledge, beliefs, and practices of family caregivers involved in disease management (13).

Assessing caregivers' current knowledge, attitudes, and practices regarding patient care can inform the development of targeted and effective health education strategies, thereby enhancing caregivers' competencies and improving patients' quality of life. Therefore, this study investigates the caregiving status of primary caregivers of patients with post-stroke dysphagia using the KAP survey model, aiming to provide a theoretical basis for future caregiver management and intervention strategies in this population.

## 2 Participants and methods

### 2.1 Participants

A cross-sectional survey was conducted using convenience sampling among eligible participants from the departments of neurology and rehabilitation at a tertiary hospital in Jiangsu Province. Inclusion criteria for patients were as follows: (1) diagnosed with stroke and exhibiting dysphagia; (2) a Functional Oral Intake Scale score of level 2 or above (14); and (3) dependent on others for feeding assistance. Inclusion criteria for primary caregivers were as follows: (1) aged 18 years or older; (2) the main family caregiver, not in a paid caregiving role; (3) continuously provided care for more than 1 week; (4) possessed normal cognitive and comprehension abilities, as determined by the trained clinical staff based on their ability to understand the study's purpose and procedures, and to provide informed consent; and (5) informed consent and voluntary participation in the study. Exclusion criteria for patients included (1) presence of severe comorbid conditions (e.g., organ failure); (2) patients requiring parenteral nutrition; and (3) patients fully dependent on enteral tube feeding. Exclusion criteria for caregivers were illiteracy or severe hearing impairment that prevented them from completing the survey. According to the rule of thumb that the sample size should be 5–10 times the number of questionnaire items, and accounting for a 20% attrition rate, a total of 286 questionnaires were distributed for this study (15).

### 2.2 Instruments

#### 2.2.1 General information questionnaire

Demographic and clinical data were collected, including the patient's age, sex, and dysphagia treatment, as well as the caregiver's age, sex, education level, caregiving duration, and employment status.

#### 2.2.2 Investigation tools

The KAP Questionnaire for Primary Caregivers of Patients with post-stroke dysphagia was developed using the Delphi method. This process involved two rounds of consultations with a panel of 10 experts from six tertiary hospitals in China, specializing in clinical nursing, nursing management, and nursing research. Under the guidance of our supervisor, we provided the experts with a detailed introduction to the study's objectives and background, along with a consultation form to gather their opinions. The questionnaire items were originally developed in Chinese and were revised based on the experts' feedback and ratings until a consensus was reached. To ensure the scientific validity and reliability of the questionnaire, we assessed expert engagement, authority, and the coordination of their opinions. The expert engagement rate was 100%, with an average authority score of 0.875. The results from the first round of consultation showed a coefficient of variation (CV) ranging from 0.08 to 0.35, and the second round yielded a CV between 0 and 1.5. The final questionnaire consists of 28 items across three dimensions. (1) Knowledge dimension: this section consists of 16 true-or-false items. Each correct answer is scored as 1 point, whereas incorrect or “uncertain” responses are scored as 0. The total score ranges from 0 to 16, with higher scores indicating better knowledge of dysphagia care. (2) Attitude dimension: this section includes five items rated on a 5-point Likert scale, ranging from “strongly disagree” (1 point) to “strongly agree” (5 points), with a total score ranging from 5 to 25. Higher scores reflect more positive caregiving attitudes. (3) Practice dimension: this section includes seven items, each rated on a 5-point Likert scale from “never” (1 point) to “always” (5 points), with a total score ranging from 7 to 35. Higher scores indicate better caregiving practices. The total questionnaire score reflects the caregiver's overall KAP level, with higher scores indicating better performance. The instrument demonstrated good reliability and validity, with a Cronbach's alpha coefficient of 0.906, split-half reliability of 0.939, and scale-level content validity index of 0.95. These results support the questionnaire's suitability for assessing the KAP status of primary caregivers of patients with post-stroke dysphagia.

Dysphagia treatment refers to whether the patient has previously received interventions for post-stroke dysphagia. The interventions considered as “treatment” include rehabilitation training and dietary adjustments for safe eating. Rehabilitation training encompasses oral muscle exercises, pharyngeal sensory stimulation (including ice and sour stimulation), compensatory swallowing techniques (e.g., dry swallowing, chin-tuck swallowing, and lateral swallowing), as well as coughing and breathing exercises. Dietary adjustments involve selecting food textures, optimizing eating posture and environment, and employing appropriate feeding techniques.

Functional ability refers to the level of dependency in daily activities and is assessed using the Barthel Index, a standardized evaluation scale. In this study, functional ability is classified as follows: a score of 100 indicates complete independence, scores of 60–99 indicate mild dependency, scores of 41–59 indicate moderate dependency, and scores of 40 or below indicate severe dependency. The Barthel Index evaluates various activities, including eating (0–10 points), bathing (0–5 points), grooming (washing face, brushing teeth, etc., 0–5 points), dressing (0–10 points), bowel control (0–10 points), bladder control (0–10 points), toileting (0–10 points), transferring from bed to chair (0–15 points), walking on level ground (0–15 points), and climbing stairs.

Health status refers to the caregiver's subjective assessment of the patient's overall health condition without using standardized measurement scales. In this study, caregivers were asked to provide a general self-evaluation of the patient's health status through open-ended responses.

#### 2.2.3 Data collection

Blinding was not employed during data collection. All investigators received standardized training prior to data collection, focusing on ensuring correct questionnaire administration, accurate data recording, and maintaining participant autonomy. During questionnaire completion, investigators could provide instructions, answer procedural questions (e.g., how to fill out a specific item, where to find sections), and ensure participants completed the questionnaires independently and privately, without external discussion or assistance. Participants were explicitly instructed to refrain from discussing their responses with others during the process. Upon completion, all questionnaires were collected on-site and managed by the principal investigator/a designated research coordinator. This individual was responsible for ensuring all questionnaires were returned and securely stored to maintain data integrity and maximize the response rate. Quality control measures included full-time supervision by investigators during the questionnaire completion process. To ensure data quality, real-time clarification was provided by investigators for any questions or difficulties encountered by participants. Questionnaires with uniform answers across all items or with more than 5% of items missing were considered invalid and excluded from the final analysis. All data were entered using a double-entry verification process conducted by two independent data entry personnel. These personnel were trained research assistants who were not involved in the data collection process, ensuring their independence. Each entry was cross-verified by the second person to identify and rectify any discrepancies before finalizing the dataset.

#### 2.2.4 Statistical analysis

All statistical analyses were performed using SPSS26.0. The Kolmogorov–Smirnov test was used to assess the normality of continuous variables. Normally distributed data were expressed as mean ± standard deviation (*x* ± *s*). For the comparison of means between two related samples, a paired-sample *t*-test was used; for independent samples, an independent samples *t*-test was employed. One-way analysis of variance was used for comparisons among multiple groups. Categorical data were expressed as frequencies (*n*) or percentages (%). The chi-square (χ^2^) test was used for group comparisons when applicable; otherwise, Fisher's exact test was applied. Multivariate analysis was performed using multiple regression analysis, with categorical variables entered as dummy variables. A two-sided *P*-value of < 0.05 was considered statistically significant.

## 3 Results

### 3.1 Baseline characteristics of patients and their primary caregivers

A total of 268 valid questionnaires were included in the final analysis. The baseline demographic and clinical characteristics of the stroke patients with dysphagia and their primary caregivers are summarized in Table 1. The mean age of the patients was 66.36 ± 8.71 years, with a majority being men (59.0%). Most patients (80.6%) presented with post-stroke sequelae, and less than half (42.9%) had received specific dysphagia treatment. In terms of functional independence in daily activities, as measured by the Barthel Index, 58.6% of patients were moderately dependent.

**Table 1:** Baseline characteristics of stroke patients with dysphagia and their primary caregivers (*n* = 268).

**Characteristic**	**Category**	***n* (%) or Mean ±SD**
**Patient characteristics**		
Age (years)		66.36 ± 8.71
Sex	Male	158 (59.0)
	Female	110 (41.0)
Post-stroke sequelae	Present	216 (80.6)
	Absent	52 (19.4)
Dysphagia treatment received	Yes	115 (42.9)
	No	153 (57.1)
Functional ability (Barthel Index)	Mild dependence (60–99)	85 (31.7)
	Moderate dependence (41–59)	157 (58.6)
	Severe dependence ( ≤ 40)	26 (9.7)
**Primary caregiver characteristics**		
Age (years)		53.82 ± 11.24
Sex	Male	89 (33.2)
	Female	179 (66.8)
Relationship to patient	Spouse	136 (50.7)
	Child	104 (38.8)
	Others	28 (10.4)
Education level	Elementary school or lower	92 (34.3)
	Junior high school	68 (25.4)
	Senior high/ Secondary technical school	70 (26.1)
	Junior college or higher	38 (14.2)
Residence	Urban	105 (39.2)
	Rural	163 (60.8)
Monthly household income (RMB)	≤ 1,000	37 (13.8)
	1,001–3,000	94 (35.1)
	3,001–5,000	84 (31.3)
	>5,000	53 (19.8)
Employment status	Unemployed	58 (21.6)
	Company employee	57 (21.3)
	Farmer	68 (25.4)
	Laborer	53 (19.8)
	Institution employee	32 (11.9)
Self-reported health status	Good	134 (50.0)
	Fair	111 (41.4)
	Poor	23 (8.6)
Caregiving duration	≤ 1 month	124 (46.3)
	1–3 months	103 (38.4)
	>3 months	41 (15.3)
Professional caregiving assistance	Yes	122 (45.5)
	No	146 (54.5)

Regarding the primary caregivers, their mean age was 53.82 ± 11.24 years, with a predominance of women (66.8%). Spouses constituted the largest relationship group (50.7%), followed by children (38.8%). A substantial proportion of caregivers (59.7%) had an educational level of junior high school or below. The caregiving duration varied, with 46.3% having provided care for over 1 month. The monthly household income was distributed across different levels, with 31.3% reporting an income between 3,001 and 5,000 RMB.

### 3.2 Knowledge, attitude, and practice status of primary caregivers for patients with post-stroke dysphagia

A total of 286 questionnaires were distributed, of which 268 valid questionnaires were returned after excluding invalid responses, yielding a valid response rate of 94%. The knowledge dimension had a total possible score of 16, with a mean score of 7.30 ± 4.07. The attitude dimension had a total score of 25, with a mean score of 19.70 ± 3.49. The practice dimension had a total score of 35, with a mean score of 19.56 ± 5.22. Item-level scores for each KAP dimension are shown in Table 2.

**Table 2:** Primary caregivers' scores for knowledge, attitudes, and practices.

**Dimension**	**Item**	**Mean Score**	**Ranking**
Knowledge	Is dysphagia caused by damage to swallowing-related neural pathways following cerebral infarction?	0.71	Top 3
	Is homogeneous, cohesive, puree-textured food beneficial for swallowing?	0.71	
	Is frequent coughing or choking while drinking a key symptom of post-stroke dysphagia?	0.66	
	Can coughing and respiratory exercises effectively prevent aspiration?	0.25	Bottom 3
	Can compensatory swallowing strategies (dry swallow, chin tuck, head turn) help clear pharyngeal residue?	0.18	
	Can oropharyngeal sensory stimulation (e.g., cold or sour stimuli) enhance the swallowing reflex?	0.18	
Attitude	Do you believe it is necessary to increase attention to the patient's swallowing function?	4.5	Top 2
	As a caregiver, is it important to learn and master dysphagia care knowledge and skills?	4.1	
	Do you think acquiring dysphagia care knowledge and skills can reduce complications like aspiration and pneumonia?	3.71	Bottom 2
	Do you believe that preventing aspiration and pneumonia requires involvement from both medical staff and family caregivers?	3.46	
Practice	Do you assess the patient's physical condition before meals to determine if feeding is appropriate?	4.09	Top 3
	Do you observe the patient's breathing status during meals?	3.53	
	Do you perform oral hygiene for the patient after each meal?	3.07	
	Can you assist the patient in clearing pharyngeal residue using compensatory swallowing techniques after meals?	2.31	Bottom 3
	Do you help the patient with basic swallowing rehabilitation exercises?	2.11	
	Do you actively seek dysphagia-related information through books or online resources?	1.93	

### 3.3 Influence of caregiver and patient demographics on knowledge, attitude, and practice score

Table 2 presents the KAP scores of primary caregivers based on various demographic factors of both caregivers and patients. Regarding patient demographics, the presence of post-stroke sequelae significantly impacted caregivers' knowledge scores (*P* = 0.023); caregivers involved in dysphagia treatment exhibited markedly higher knowledge (*P* = 0.001) and practice scores (*P* = 0.001) compared with those not involved in such treatment. In terms of caregiver demographics, age emerged as a critical factor, with younger caregivers ( ≤ 50 years) showing significantly higher KAP scores across all measures (*P* < 0.001). Furthermore, educational attainment was strongly correlated with KAP scores, where caregivers with higher education levels (junior college or higher) scored significantly better in knowledge, attitudes, and practices (*P* < 0.001). Moreover, caregivers who were children of the patients scored higher in knowledge and practice compared with spouses or other relatives (*P* < 0.001). Further details are provided in Table 3.

**Table 3:** Influence of caregiver and patient demographics on knowledge, attitude, and practice score (*n* = 268, *x*±*s*).

**Participants**	**Variable**	**Category**	***n***	**Knowledge**	**Attitude**	**Practice**
Patient	Age (years)	≤ 50	6	9.00 ± 2.19	22.83 ± 1.33	22.50 ± 1.64
		51–60	67	8.15 ± 4.18	19.60 ± 3.27	20.15 ± 5.76
		61–70	99	6.45 ± 3.92	19.28 ± 3.67	18.95 ± 5.27
		71–80	86	7.49 ± 4.24	20.22 ± 3.48	20.67 ± 4.86
		≥81	10	7.50 ± 2.84	18.20 ± 2.82	20.90 ± 3.96
	*t/F*-value			2.149	2.574	1.812
	*P*-value			0.075	0.038	0.127
	Sex	Male	158	7.46 ± 3.89	19.78 ± 3.43	20.35 ± 5.08
		Female	110	7.09 ± 4.32	19.58 ± 3.60	19.39 ± 5.38
	*t/F*-value			0.721	0.467	1.481
	*P*-value			0.471	0.641	0.140
	Daily activities	Mild dependence	85	7.75 ± 4.17	19.94 ± 3.44	20.32 ± 5.66
		Moderate dependence	157	7.10 ± 4.11	19.67 ± 3.47	20.02 ± 5.02
		Heavy dependence	26	7.08 ± 3.50	19.12 ± 3.86	18.38 ± 4.78
	*t/F*-value			0.750	0.571	1.399
	*P*-value			0.473	0.565	0.249
	Post-stroke Sequelae	Present	216	7.58 ± 4.02	19.78 ± 3.46	20.19 ± 5.05
		Absent	52	6.15 ± 4.11	19.37 ± 3.62	19.00 ± 5.83
	*t/F*-value			2.293	0.772	1.474
	*P*-value			0.023	0.441	0.142
	Dysphagia treatment	Present	115	8.23 ± 4.45	19.52 ± 3.68	21.15 ± 5.82
		Absent	153	6.61 ± 3.62	19.84 ± 3.35	19.06 ± 4.53
	*t/F*-value			3.268	−0.730	3.304
	*P*-value			0.001	0.466	0.001
	Type of medical insurance coverage	Out-of-pocket	48	8.04 ± 4.26	19.44 ± 3.43	20.40 ± 5.25
		Urban employee basic medical insurance	154	7.23 ± 4.20	20.60 ± 3.28	20.10 ± 5.29
		New rural cooperative medical scheme		6.95 ± 3.58	17.79 ± 3.25	19.29 ± 5.03
	*t/F*-value			1.060	17.009	0.772
	*P*-value			0.348	< 0.001	0.463
Caregiver	Age (years)	≤ 50	77	9.64 ± 3.39	21.55 ± 2.56	22.12 ± 2.99
		51–60	111	7.69 ± 4.10	19.89 ± 3.36	20.89 ± 5.73
		61–70	73	4.70 ± 2.88	17.70 ± 3.36	16.70 ± 4.62
		71–80	7	2.71 ± 1.80	17.29 ± 4.19	15.29 ± 3.15
	*t/F*-value			28.419	19.859	20.701
	*P*-value			< 0.001	< 0.001	< 0.001
	Sex	Male	89	6.72 ± 4.25	19.09 ± 3.59	17.89 ± 4.75
		Female	179	7.60 ± 3.96	20.01 ± 3.41	20.98 ± 5.15
	*t/F*-value			−1.671	−2.034	−4.756
	*P*-value			0.096	0.043	< 0.001
	Marital status	Married	253	7.26 ± 4.00	19.63 ± 3.49	19.91 ± 5.14
		Unmarried	5	6.60 ± 4.04	20.20 ± 3.83	17.40 ± 5.94
		Divorced	10	8.80 ± 5.81	21.2 ± 3.29	22.50 ± 6.47
	*t/F*-value			0.764	1.021	1.812
	*P*-value			0.467	0.362	0.165
	Residence	Urban	105	7.40 ± 4.04	18.01 ± 3.59	20.06 ± 5.38
		Rural	163	7.25 ± 4.09	20.79 ± 2.96	19.89 ± 5.12
	*t/F*-value			0.303	−6.899	0.256
	*P*-value			0.762	< 0.001	0.798
	Education level	Elementary school or lower	92	4.76 ± 3.15	17.57 ± 3.46	16.45 ± 3.85
		Junior high school	68	6.00 ± 3.21	19.00 ± 3.28	18.68 ± 5.13
		Senior high school or secondary technical school	70	10.11 ± 3.39	21.83 ± 2.01	24.01 ± 4.08
		Junior college or higher	38	10.63 ± 3.06	22.21 ± 2.06	23.26 ± 2.29
	*t/F*-value			54.198	39.528	55.588
	*P*-value			< 0.001	< 0.001	< 0.001
	Relationship to patient	Spouse	136	6.28 ± 3.79	18.73 ± 3.53	18.66 ± 5.33
		Child	104	8.83 ± 3.72	20.88 ± 3.03	21.73 ± 3.88
		Others	28	6.64 ± 4.92	20.04 ± 3.60	19.64 ± 7.00
	*t/F*-value			13.051	12.349	11.022
	*P*-value			< 0.001	< 0.001	< 0.001
	Professional caregiving assistance	Present	122	7.19 ± 4.31	19.89 ± 3.54	20.11 ± 5.47
		Absent	146	7.40 ± 3.87	19.54 ± 3.46	19.83 ± 5.01
	*t/F*-value			−0.431	0.822	0.433
	*P*-value			0.667	0.412	0.665
	Monthly household income	≤ 1,000	37	4.16 ± 3.41	17.38 ± 3.87	16.35 ± 6.03
		1,001–3,000	94	6.31 ± 3.84	18.36 ± 3.36	18.61 ± 4.77
		3,001–5,000	84	7.93 ± 3.54	20.86 ± 2.77	21.02 ± 4.69
		>5,000	53	10.28 ± 3.49	21.87 ± 2.42	23.17 ± 3.70
	*t/F*-value			24.473	25.401	19.084
	*P*-value			< 0.001	< 0.001	< 0.001
	Employment status	Between jobs	135	7.30 ± 4.24	19.75 ± 3.54	19.93 ± 5.05
		In-service	133	7.32 ± 3.91	19.65 ± 3.46	19.98 ± 5.40
	*t/F*-value			−0.039	0.220	−0.069
	*P*-value			0.969	0.826	0.945
	Employment status	Unemployed	58	5.21 ± 3.28	18.84 ± 3.52	18.26 ± 5.61
		Company employee	57	9.74 ± 3.02	21.67 ± 2.13	22.33 ± 2.86
		Farmer	68	4.28 ± 3.24	17.49 ± 3.69	16.49 ± 5.09
		Laborer	53	8.34 ± 3.28	19.66 ± 2.81	20.96 ± 4.27
		Institution employee	32	11.50 ± 2.54	22.53 ± 1.95	24.50 ± 3.16
	*t/F*-value			46.683	23.339	25.358
	*P*-value			< 0.001	< 0.001	< 0.001
	Self-reported health status	Good	134	7.46 ± 3.91	20.01 ± 3.59	20.49 ± 5.02
		Fair	111	7.18 ± 4.00	19.41 ± 3.18	19.40 ± 5.07
		Poor	23	7.00 ± 5.33	19.30 ± 4.30	19.52 ± 6.74
	*t/F*-value			0.216	1.038	1.431
	*P*-value			0.806	0.355	0.241
	Caregiving duration	≤ 1 month	124	6.15 ± 3.92	19.90 ± 3.55	18.70 ± 5.72
		1–3 months	103	7.56 ± 3.99	19.45 ± 3.67	20.35 ± 4.66
		>3 months		10.14 ± 3.17	19.73 ± 2.86	22.76 ± 3.51
	*t/F*-value			16.995	0.481	10.477
	*P*-value			< 0.001	0.619	< 0.001

### 3.4 Linear regression analysis of factors influencing caregivers' knowledge, attitude, and practice scores

The total KAP score was used as the dependent variable. Independent variables included those found to be statistically significant in the univariate analysis (i.e., variables showing significance in at least one of the three dimensions: knowledge, attitude, or practice). Stepwise regression was applied for variable inclusion and removal (α_entry_ = 0.05, α_removal_ = 0.10), and variables with significant interaction effects were excluded. Multiple linear regression analysis was conducted using the variable coding scheme, as shown in Table 4. The regression results are presented in Table 5.

**Table 4:** Coding scheme for independent variables.

**Variable**	**Coding description**
**Patient characteristics**	
Patient age	≤ 50 = 1; 51–60 = 2; 61–70 = 3; 71–80 = 4; ≥81 = 5
Post-stroke sequelae	Present = 1; Absent = 2
Dysphagia treatment	Present = 1; Absent = 2
Type of medical insurance coverage	Out-of-pocket = 1; Urban Employee Basic Medical Insurance = 3; New Rural Cooperative Medical Scheme = 4
**Primary caregiver characteristics**	
Caregiver's age, ≤ 50 = 1; 51–60 = 2; 61–70 = 3; 71–80 = 4	
Caregiver's sex	Male = 1; Female = 0
Residence	Rural = 1; Urban = 2
Education level	Elementary school or lower = 1; Junior high school = 2; Senior high school or secondary technical school = 3; Junior college or higher = 4
Relationship to patient	Spouse = 1; Child = 2; Others = 3
Employment status	Unemployed = 0; Company employee = 1; Farmer = 2; Laborer = 3; Institution employee = 4
Monthly household income	≤ 1000 = 1; 1001–3000 = 2; 3001–5000 = 3; >5000 = 4
Caregiving duration	≤ 1 month = 1; 1–3 months = 2; >3 months = 3

**Table 5:** Multiple linear regression analysis of factors influencing caregivers' knowledge–attitude–practice scores.

**Variable**	**Unstandardized coefficient**		**Standardized coefficient**	***t-*value**	***P*-value**	**Variance inflation factor**
	***B***	**SE**	β			
(Constant)	31.428	3.188		9.858	< 0.001	
Education level	4.892	0.582	0.485	8.399	< 0.001	2.076
Caregiving duration	4.120	0.603	0.277	6.834	< 0.001	1.022
Monthly household income	1.995	0.562	0.178	3.548	< 0.001	1.568
Dysphagia treatment	−2.337	0.876	−0.108	−2.669	0.008	1.017
Caregiver's age	−1.795	0.729	−0.136	−2.463	0.014	1.903

## 4 Discussion

### 4.1 Current status of knowledge, attitudes, and practices among primary caregivers of patients with post-stroke dysphagia

In this study, the mean age of patients with post-stroke dysphagia was 66.36 ± 8.71 years, consistent with the known increase in cerebrovascular disease risk with advancing age (16), as well as the previously confirmed positive correlation between age and the incidence of dysphagia (17). In terms of sex distribution, a higher proportion of male patients was observed. This aligns with the findings obtained by Li et al. (17), who reported a higher incidence of post-stroke dysphagia in men. Most primary caregivers were either the spouse or children of the patient, with women accounting for 66.8% of caregivers. This is in line with the findings obtained by Liu et al. (18) and may reflect the influence of traditional cultural norms in China, where women are typically expected to manage domestic responsibilities. Additionally, personality traits such as attentiveness may make women more suited to caregiving roles compared with men. Regarding educational background, 59.7% of caregivers had only primary or junior secondary education. This is consistent with the survey conducted by Zhang (19) on home caregivers of patients with stroke in Zhengzhou. One possible explanation for this finding is that most of the caregivers in this study were middle-aged individuals, a generation for whom access to education was relatively limited, resulting in generally lower educational attainment.

Our study results showed that the mean score for caregiving knowledge among primary caregivers of patients with post-stroke dysphagia was 7.30 ± 4.07 out of a total of 16, indicating a moderate to low level of knowledge. This finding is consistent with those reported in previous studies (20). In the knowledge dimension, caregivers scored lowest on items related to dysphagia rehabilitation training, suggesting an inadequate understanding of this aspect of care. Similar findings were reported by Li et al. (21), who found that almost 50% of both patients and caregivers lacked knowledge of how to conduct rehabilitation training. Jiang et al. (22) also observed that caregivers often had limited knowledge of stroke rehabilitation, lacked the ability to perform basic training, and expressed a desire for professional guidance. These findings are consistent with the present study. A possible explanation for this lack is that dysphagia rehabilitation involves specialized techniques, and many healthcare professionals have limited training in this area. As a result, caregiver education in clinical settings is often superficial and insufficient. The inadequate caregiver knowledge observed in this study contradicts expert consensus guidelines, which recommend that family caregivers be actively involved in post-stroke rehabilitation. These findings highlight the urgent need to enhance caregivers' awareness and understanding of dysphagia rehabilitation. Healthcare professionals should prioritize targeted education and training to equip caregivers with the essential knowledge and skills needed to support effective rehabilitation at home.

The study results showed that the mean score for caregiving attitude among primary caregivers of patients with post-stroke dysphagia was 19.70 ± 3.49 out of a total of 25, indicating a moderately high level of caregiving attitude. This aligns with the survey results reported by Liu et al. (23). An item-level analysis of the attitude dimension revealed that the question “Do you think acquiring dysphagia care knowledge and skills can reduce complications like aspiration and pneumonia?” received relatively low scores. Previous studies (24) demonstrated that caregivers equipped with appropriate dysphagia care knowledge and skills can significantly improve patients' nutritional intake and feeding status, enhance quality of life, and reduce complications such as aspiration, choking, and pneumonia. However, our findings suggest that many caregivers do not fully recognize the impact of their own caregiving knowledge and skills on patient health outcomes. This might be explained by the fact that clinical health education efforts often focus primarily on the patients themselves, with less attention given to family caregivers. As a result, caregivers may lack awareness of their role in recovery and may not understand how their level of caregiving competence can influence patient prognosis.

The mean score for caregiving practices among primary caregivers of patients with post-stroke dysphagia was 19.56 ± 5.22 out of a possible 35, indicating a moderate to low level of caregiving practices. This finding is consistent with the results reported by McLennon et al. (25), who found that 75% of primary caregivers of patients with stroke lacked proper training, exhibited poor caregiving skills, and had unmet caregiving needs. Notably, the item “Do you actively seek dysphagia-related information through books or online resources?” received a particularly low score. This may be attributed to the fact that most caregivers in this study had only a primary or junior secondary education, which may limit their initiative or ability to seek disease-related information independently. Items such as “Can you assist the patient in clearing pharyngeal residue using compensatory swallowing techniques after meals?” and “Do you perform oral hygiene for the patient after each meal?” also scored poorly. This indicates that many caregivers lack awareness of the importance of removing oral residue and maintaining oral hygiene, consistent with the results obtained by Duan et al. (26), who reported that 69.4% of caregivers were unaware of proper oral care procedures. Similarly, the item “Do you assist the patient in performing basic swallowing rehabilitation exercises?” scored low. This may be associated with the requirements of specialized swallowing rehabilitation, which is typically provided by speech and language therapists. Many clinical healthcare providers themselves lack training in swallowing therapy techniques (27, 28), which may result in vague or insufficient guidance being provided to caregivers. Consequently, caregivers may struggle to understand or apply rehabilitation methods effectively. These findings suggest that to improve caregivers' engagement in the rehabilitation process, clinical healthcare professionals should not only educate them on specialized feeding techniques for dysphagia but also strengthen instruction on rehabilitation methods.

### 4.2 Influence of sociodemographic factors on caregivers' knowledge, attitudes, and practices

In this study, education level, caregiving duration, caregiver age, and whether the patient received dysphagia treatment were identified as significant factors influencing caregivers' KAP scores. Both age and education level are closely associated with an individual's ability to comprehend and adapt to new information. Previous studies have found that caregivers with higher educational attainment are more attentive to dysphagia care and treatment, more capable of understanding and accepting disease-related knowledge, and more likely to obtain relevant information through various channels (20). This study also showed that longer caregiving duration was associated with improved knowledge and changes in attitude and practices, consistent with prior findings (20). This is primarily because caregivers in the early stages of care tend to focus on acute symptoms such as cognitive or mobility impairments, paying less attention to swallowing difficulties. However, over time, as caregiving continues, they accumulate practical experience and gain a deeper understanding of caregiving knowledge and skills. Additionally, whether the patient received dysphagia rehabilitation notably affected caregiver KAP outcomes. Patients and caregivers who were involved in dysphagia treatment had greater opportunities to learn about rehabilitation strategies and techniques, providing caregivers with a foundational understanding to implement these methods during daily care. Supporting this, Eltringham et al. (29) found that effective communication during dysphagia rehabilitation enabled patients and their caregivers to better understand the pathophysiology of post-stroke dysphagia and the principles of rehabilitation therapy. In addition, a previous survey (27) revealed that many nurses lack training in dysphagia rehabilitation techniques, with 96% unaware of the role of speech–language pathologists in managing dysphagia. This deficiency may contribute to caregivers' inadequate understanding and application of dysphagia rehabilitation training. While structured training programmes on dysphagia management for healthcare professionals have been implemented in some countries, such initiatives remain limited in China. Therefore, hospitals should prioritize professional development and training for clinical staff in dysphagia rehabilitation methods.

### 4.3 Study limitations

This study found that while caregivers of patients with post-stroke dysphagia exhibited relatively positive attitudes, their knowledge and caregiving practices remained insufficient. Considering that patients with stroke often experience reduced ability to perform daily activities, caregivers serve as their primary source of support. As such, the KAP level of caregivers plays a crucial role in the comprehensive management of post-stroke dysphagia. Healthcare professionals should develop individualized and targeted health education interventions based on the identified influencing factors of KAP. Such interventions could enhance caregivers' KAP levels and caregiving competence, reduce their burden, and ultimately promote better rehabilitation outcomes for patients.

## 5 Conclusion

In conclusion, while primary caregivers of patients with post-stroke dysphagia demonstrated moderately positive attitudes, their caregiving knowledge and practices require improvement. Key factors influencing caregivers' KAP scores include education level, caregiving duration, caregiver age, whether the patient received dysphagia treatment, and monthly household income.

## Data Availability

The original contributions presented in the study are included in the article/supplementary material, further inquiries can be directed to the corresponding author.
